# Main actors in the new population policy with a growing trend in Iran: a stakeholder analysis

**DOI:** 10.1186/s41043-022-00338-2

**Published:** 2022-12-12

**Authors:** Hasan Jafari, Abolghasem Pourreza, Neda Kabiri, Rahim Khodyari-Zarnaq

**Affiliations:** 1 Department of Health Care Management, Health Policy and Management Research Center, School of Public Health, Shahid Sadoughi University of Medical Sciences, Yazd, Iran; 2 Department of Management and Health Economics, School of Public Health, Tehran University of Medical Science, Tehran, Iran; 3 Research Center for Evidence-based Medicine, Tabriz University of Medical Sciences, Tabriz, Iran; 4 Department of Health Policy and Management, Iranian Center of Excellence in Health Management, School of Management and Medical Informatics, Tabriz University of Medical Sciences, Tabriz, Iran; 5 Tabriz Health Service Management Research Center, School of Management and Medical Informatics, Tabriz University of Medical Sciences, Tabriz, Iran; 6 Research Center of Psychiatry and Behavioral Sciences, Tabriz University of Medical Sciences, Tabriz, Iran

**Keywords:** Policy analysis, Stakeholder analysis, Population policy, Total fertility rate, Islamic Republic of Iran

## Abstract

The total fertility rate in Iran has declined to below replacement level recently, and a new approach has been taken to tackle this issue. Thus, this study aimed to identify the involved stakeholders and their characteristics in the new population policy change in Iran. We employed a qualitative approach using the purposive sampling of key informants and the identification of relevant documents. The main stakeholders were divided into seven key groups: religious, political, governmental, professional, international sectors, media, and nongovernmental organizations. In addition, there was no centralized, clear, and comprehensive mechanism to guide the activities of stakeholders to coordinate and bring the total fertility rate to the replacement level in Iran. Despite the importance of the new population policy in Iran, in recent years, we still experience dispersion and inconsistency among various actors in this area. It is imperative to go through a consensus and coalition at macro-level authorities alongside evidenced-based population policymaking.

## Introduction

Population policy is highly complex and intensely political and directly linked to a country's socioeconomic development [1]. In recent years, particularly in the last decade of the twentieth century, most countries have experienced a decline in the total fertility rate (TFR). Of course, there have been significant differences in the TFR decline between various countries [2]. Developing countries, similar to other countries in the world, have experienced a quick decline in the TFR in recent decades [3].

The Islamic Republic of Iran is a developing country of TFR = 2.01 live births per woman according to the last census of 2016 [4] and is moving toward a growing old population. Such a low population growth rate and demographic transition (total fertility rate of 1.92—less than the replacement fertility level) and their potential socioeconomic consequences have driven the shift in policies [5]. It forced executives and policymakers of Iran to review the former population policy (which has been mainly focused on birth control via family planning) and develop a new policy to avoid the aging problem in the future [6].

The fertility transition in Iran has some key features before and after the Islamic Revolution in 1978. As the first stage, there was a reduction in the fertility rate in the early 1970 after a long period of high fertility. In the second stage (1976–1980), fertility increased, and it was relatively stable until 1984 (third stage). The fertility rate decreased slowly from 1985 to 1988 (fourth stage) and dramatically declined from 1989 until 2012 (fifth stage) [7]. The predicted future situation of a reduced young population led to the new population policy aiming to support childbearing in 2014 (sixth stage) [8, 9].

Results of a systematic review indicated that there are five main factors for the declining trend in TFR in the Middle East, which include healthcare-related, social, cultural, economic, and political. It is necessary to identify these factors in a country with declining TFR and consider them in population policies [10]. There are some policies to support the goals of the new population policy, for example, restriction of access to family planning services, increasing maternity leave from 3 months to 9 months, and promoting natural childbirth [11, 12].

To understand the roles, interests, and powers of the different stakeholders in the evolution of policy context and processes, stakeholder analysis (SHA) is a powerful tool [13]. Using the information gathered from SHA can develop strategies for collaborating with stakeholders, facilitating the implementation of choices or organizational objectives, and then, understanding the future of policy directions [14].

In the current study, we used three key steps followed by Roberts et al.’s work [15] to analyze stakeholders of the new population policy in Iran. The main reason for using this approach is that it adopts an analytical approach to health reform and is recommended for policymakers and practitioners involved in the design, execution, and evaluation of healthcare reforms. The three steps to this approach include firstly, identifying the stakeholders relevant to the policy of interest, secondly, determining the current stakeholders' position (in terms of support, opposition, or non-mobilized), and thirdly, assessing the relative power of each stakeholder (in terms of high, medium, or low).

Since there were many different pros and cons to the Iranian new population policy, this study aimed to identify the involved stakeholders and their characteristics in terms of position and level of power in this policy to manage stakeholders and identify opportunities to mobilize their support for this particular goal.

## Methods

### Approach

A study is a qualitative approach conducted at a national level in 2017, in the Islamic Republic of Iran.

### Participants and recruitment of key informants

A purposeful sampling method was used to identify key informants. Individuals or organizations that have specific experience or knowledge in the area of population, reproductive health, and population policymaking and other high-level authoritative executives were recruited in the study. In the following, we also employed network sampling [16] to recognize other key informants (KIs) until reaching saturation.

### Selecting and validating the policy documents

Thirty related documents were collected in kinds of upstream documents (like issued policies by the supreme leader, 5-year economic, social, and cultural plans of development), acts and rules by parliament, plans and programs by government, and national and international reports. It is noteworthy that the majority of documents had high value and acceptability.

### Data collection

All semi-structured and face-to-face interviews were carried out by one member of the study team (HJ). The interviews lasted an average of 60 min. The interviews were conducted in the interviewee's office or any place where the participant suggested. Recorded interviews were then transcribed verbatim. Data saturation occurred after 18 interviews.

### Data management and analysis

Framework analysis, which is a qualitative method and aptly suited for applied policy research, is used to analyze the data. The step-by-step guide proposed by Braun and Clarke [17] including familiarizing with data, generating initial codes, searching for themes, reviewing themes, defining and naming themes, and producing the report was by the MAXQDA software, Version 12. Data analysis was performed simultaneously and in parallel with interviews. Additionally, we applied Policy Maker 4 software to identify all the stakeholders, the affected, involved, and influential and assess the position and level, and power of the stakeholders regarding policy change.

### Quality and rigor of the study

For more credibility, we tried to use the maximum diversity approach for selecting participants from various governmental, legislative, nongovernmental, and international institutions. In addition, triangulation such as the methods of collection (interview, observation of writing, and observation of documents) was used to obtain better results. After each review, the original audio was transcribed verbatim and sent to the participant for comment.

One member of the research team (HJ) conducted all of the interviews. This bias prevention strategy increased a large extent the accuracy of work and greater coordination. Continual attendance of the interviewer in all interviews and spending enough time for more accurate data, as well as clarity of methodology, added transformability.

### Ethical consideration

The present study received ethical approval from the ethics committee of the Tehran University of Medical Sciences (Research Ethics Committee Reference: 9021460003). The investigators obtained the participants' permission to perform and audiotape the interviews. The confidentiality of information was guaranteed, as the name and personal information of the interviewees were not stated in the tapes or transcripts. All tapes, transcripts, and information sheets were given special codes and kept distinctly to protect the participants' anonymity.

## Results

We included the results from the review of relevant documents and the interviews with key informants. These findings encompass identifying the stakeholders and their related sectors, positions, and levels of power of stakeholders for the new population policy in Iran.

In total, 18 participants were included in the study from different organizations including the Health Commission of the Islamic Parliament [Majles-e-Shoray-e-Eslami]; Office of population, family and schools health at the Ministry of Health, Treatment and Medical Education (MoHME); National Organization for Civil Registration (NOCR); Institute for Research (IR); Statistical Center of Iran (SCR); United Nations Population Fund (UNFPA); Non-Governmental Organizations (NGOs) in the field of the population; and Academics (demography and population and reproductive health professors and researchers in the field) (Table 1).

**Table 1:** Characteristics of participants

Participant	Position	Organization	Sex	Academic degree
Participant 1	Top manager	More	Male	MD
Participant 2	Senior expert	UNFPA	Female	MD
Participant 3	Top manager	IR	Female	PhD
Participant 4	Senior expert	NGO	Female	MD
Participant 5	Parliamentarian	Parliament	Male	MD
Participant 6	Associate professor	Academics	Male	PhD
Participant 7	Associate professor	Academics	Female	PhD
Participant 8	Top manager	NOCR	Male	MS
Participant 9	Senior expert	UNFPA	Male	MS
Participant 10	Senior expert	MoHME	Female	BSc
Participant 11	Expert	SCI	Female	BSc
Participant 12	Senior expert	MoHME	Male	MS
Participant 13	Assistant professor	Academics	Male	PhD
Participant 14	Assistant professor	Academics	Male	PhD
Participant 15	Professor	Academics	Male	PhD
Participant 16	Top manager	NGO	Male	PhD
Participant 17	Middle manager	SCI	Male	M.S
Participant 18	Expert	NOCR	Female	BSc

Based on the findings from analyses of this study, stakeholders of the new population policy in Iran were divided into seven key sectors of religious, political, governmental, professional, international, media, and NGOs (Table 2).

**Table 2:** Stakeholders and their positions and levels of powers for new population policy in Iran

Stakeholder	Sector	Position	Power
Supreme Leader	Religious	High Support	High
Islamic Seminaries	Religious	High Support	Medium
Judiciary system	Political	Non-Mobilized	Medium
Islamic Parliament	Political	Medium Support	High
Supreme Council of the Cultural Revolution	Political	High Support	Medium
Ministry of Health	Governmental	Medium Support	Medium
Ministry of Cooperatives and Labor	Governmental	Non-Mobilized	Medium
Ministry of Education	Governmental	Non-Mobilized	Medium
Ministry of Science, Research and Technology	Governmental	Low Support	Medium
Ministry of Sport and Youth	Governmental	Low Support	Medium
Statistical Research and Training Center	Governmental	Low Support	Low
National Organization for Civil Registration	Governmental	Medium Support	Medium
Women's Affairs and Family of Presidency	Governmental	Non-Mobilized	Medium
Population Departments	Professional	Low Support	Medium
Environmental Experts	Professional	Low Opposition	Low
Islamic Republic of Iran Broadcasting	Media	High Support	High
National Population Studies & Comprehensive Management	Non-Governmental	Low Support	Low
Family Health Association of Iran	Non-Governmental	Low Support	Low
United Nations Population Fund	International	Non-Mobilized	Medium

Participants reported that a superior leader at the national level has a remarkable role and repeatedly disseminates information on the policy change, along with rationales requiring this change, and mobilized officials and the public to take up the new policy. According to the participant's perspective, the superior leader is supportive and influential, because the adoption of the new population approach and its subsequent implementation heavily depended on him.

For example, he emphasizes the importance of population issues:

One of the dangers which are frightening us by deep thinking about it is population issue. Take it seriously. The young population of the country is reducing. If several years have passed, then there are no longer curable (2014, December 20)

In addition, the overall population policies announced by the superior leader on 20/5/2014, gave a lot of political legitimacy to population growth policies, as a road map, which is considered a comprehensive document and it can somewhat determine the role of each stakeholder including the executive, legislative and judiciary institutions, NGOs, private sector and the public.

This document in the present situation is an excellent and comprehensive policy and administrative offices based on this policy should come to carry a lot of research to identify the reasons and write a detailed action plan (Participant 9).

Parliament was another stakeholder that was reportedly very influential because it was highly respected, with high support and high power on the issue. Parliamentarians proposed plans like the comprehensive plan of population and family excellence or preventing the decline of population growth as the solutions and defenders them in the mass media to raise public awareness.

At the time of our analysis, most political stakeholders were essentially non-mobilized or had low support concerning the population reform policy in Iran.

For instance, participants stated that the researcher's and population scholars' roles have not been considered, because they were not engaged in policy dialogues to clarify the possible causes and direct selection of the best choice.

Sometimes, hasty and unscientific decisions were made in population area without the use of expert opinions of demographics (for example, in Atiyeh plan) that fail (Participant 7).

In this work, environmental experts were known as a low opposition with a low power stakeholder. They have serious concerns about the issue.

The capacity of several disadvantaged provinces is low for more population. They do not have more water, land, and infrastructural capacity to accommodate more population (Participant 6).

It might be altered opposition of them by involvement in policymaking meetings and using their comments on the appropriation of the resources like water and land capacity of each region for increasing the TFR and not prescribing a unit prescription for all of the regions. Therefore, by adding desired goals or mechanisms to the policy, it might be persuaded this stakeholder to weak its opposition. In addition, meeting with opponents such as environmental experts helps to seek common goals or policies, and thereby, reduces the intensity of their opposition.

Our key informants mentioned that the role of ordinary people in policymaking is weak and largely they do not voice in governmental authorities. Namely, most decisions are made behind closed doors with a non-participatory approach.

Strategies and solutions for changing the position and power of stakeholders to increase TFR are indicated in Table 3.

**Table 3:** Solutions for changing the position and power of stakeholders

Stakeholders	Strategies
Islamic Seminaries, Supreme Council of the Cultural Revolution, Islamic Parliament, Judiciary system, Broadcasting, Ministry of Education, Ministry of Science, Research and Technology	Using media to increase the generality of the problem and change the perception of the problem
Islamic Seminaries, Ministry of Cooperatives and Labor, Ministry of Science, Research and Technology, Islamic Republic of Iran Broadcasting	Providing evidence such as technical and political information
Supreme Leader, Islamic Seminaries, Islamic Parliament, Supreme Council of the Cultural Revolution, Ministry of Health, Ministry of Cooperatives and Labor, Ministry of Science, Research and Technology, Statistical Research and Training Center, National Organization for Civil Registration, Population Departments, Family Health Association of Iran, Islamic Republic of Iran Broadcasting	Building a coalition with a specific identity with sufficient resources
Supreme Council of the Cultural Revolution, Ministry of Health, Ministry of Cooperatives and Labor, Statistical Research and Training Center, National Organization for Civil Registration, Family Health Association of Iran, Judiciary system, Ministry of Education, Ministry of Sport and Youth, Islamic Republic of Iran Broadcasting	Increasing stakeholder organizational power by providing more funding or experienced personnel
Judiciary system, Ministry of Education, Ministry of Sport and Youth, Women's Affairs and Family of Presidency, Environmental Experts, United Nations Population Fund	Encouraging stakeholders to reinforce their position by adding benefits as an incentive
Ministry of Health, Population Departments, Family Health Association of Iran, Ministry of Education, Environmental Experts, United Nations Population Fund	Encouraging stakeholders to reinforce their position by adding goals and mechanisms to policies
Ministry of Cooperatives and Labor, Ministry of Science, Research and Technology, Statistical Research and Training Center, Population Departments, Family Health Association of Iran, Judiciary system, Ministry of Education, Ministry of Sport and Youth, Women's Affairs and Family of Presidency, Environmental Experts, United Nations Population Fund	Increase access to policymakers and sources of power by forming a supportive campaign

## Discussion

In this study, the stakeholder analysis showed diverse stakeholders with different powers and positions are working on population policies in Iran. Of course, most of them are non-mobilized or had low support concerning the population reform policy. Additionally, there is no centralized, clear, and comprehensive mechanism to guide the activities of governmental, NGOs, and private organizations and institutions to coordinate and bring TFR to the replacement level.

This study led to recommendations and strategies to change the position and power of stakeholders regarding population policy in Iran. It was recommended that a coalition of supporting groups or stakeholders, with a recognizable name and sufficient resources, should be created. A steward under the supervision of the supreme leader or among all politicians including the judiciary and parliament and the presidency system should work along with NGOs to achieve the aims of the overall policy issued by the supreme leader and other politicians. For instance, the population supreme council, which was proposed by the majority of key informants, as a stewardship of the country's population policies, should be established to develop population policies, coordinate different authorities and prioritize the TFR decline issue. Civic coalitions including stakeholders are shown to be effective in making positive changes in health outcomes. When politicians build a team with different influencers and expand their partnerships, these coalitions become more effective [18].

Another strategy was adding desired goals and mechanisms to the policy based on stakeholders' ideas and suggestions (engaging all stakeholders in the policymaking process). Interventions should be founded on actuality and evidence, not on political and security perspectives. In this case, all can participate as stakeholders. As one individual stakeholder does not have all the needed skills and knowledge about the issue, the cross-disciplinary collaboration of stakeholders is essential. However, capacity building of this engagement should be done first [19]. When decisions and policies are determined without stakeholders' participation, programs could not be translated into action [20]. Despite the benefits of stakeholder engagement in the policymaking process, policymakers should be aware of the different interests of stakeholders, which may change policy positions in a way that is not based on evidence [21].

Increasing availability to political leaders, through a lobbying campaign was the next recommendation of this research to change stakeholders' positions and power. Some experts believe that the fertility decline problem is more complex and needs a high structure, not only the government but also all officials to develop and adopt policies. Donors, NGOs, and the private sector have the potential to participate in financing as well as service delivery where the government does not have enough resources [20]. Research bodies also play important role in informing policymakers and stakeholders [22].

Another strategy to change stakeholders' power and position is using the media to increase public awareness and change their perception of population issues. Of course, the above approach should be regarded as a necessary but not sufficient option along with other activities, not just that much remains conferences. Media provide information to the public at the national level and act as policy implementers; however, media are a part of political communication at the regional level [23].

Providing information and evidence to stakeholders, including technical and political information is a strategy introduced by this study that can change the position and power of stakeholders. Although the majority of respondents believed in the necessity to change the population policy, there was no consensus regarding the best alternative. Some presented evidence showing that cultural practices could be effective, while others had evidence showing the effectiveness of economic affairs. However, it seems that the participation of all stakeholders, including socioeconomic and cultural bodies is necessary and by carrying out the above strategy, the importance of the issue and its policies become higher, especially among senior authorities.

Finally, we suggest increasing the organizational strength of supporters, by providing increased material resources or experienced staff or by fostering political skills. For example, increased funding of MoHME along with other institutions by enhancing the strength of these organizations could cover the costs of infertility treatment. Technical and financial support of stakeholders has been shown to play an important role in implementing such policies in the countries [24].

## Conclusion

The results of this study indicated that because of the inter-sectoral and multidimensional nature of population policy, different stakeholders with different powers and positions are influenced by this policy in Iran. Although most of them are non-mobilized or had low support concerning the population reform policy, our results also show that the stakeholders who are in the category of high-power stakeholders have a high or medium supportive position, which can support other stakeholders through building a coalition with a specific identity and sufficient resources. Despite the importance of the new population policy in Iran, in recent years, we still experience dispersion and inconsistency among various actors in this area. It is imperative to go through a consensus and coalition at macro-level authorities alongside evidenced-based population policymaking. Based on our findings, strategies such as using media, providing evidence and information, providing more financial incentives, and forming a supportive campaign can be effective in managing the stakeholders.

## Limitations of the study

Information produced by a stakeholder analysis is always to some extent subjective and identifying stakeholders who are, or might become, involved in a particular policy process, requires the judgment of the analyst. Any analysis depends only as good as the analyst's attention, creativity, and access to the information [25]. Another limitation of this study was the sample size, although it was a conscious decision of the research team to consider qualified experts rather than a broad spectrum of participants. The consistent, in-depth, comments received in the interviews showed a suitable size of sampling. In addition, key informants may not have enough time for an interview or may simply provide official positions or what people say and how they say it, as opposed to what they do or think. For example, a minister may publicly defend a policy to win favor with voters or specific interest groups but may be actively working against it within the government. Hence, recognizing and understanding these limitations, especially in population policies, might boost the value of such studies and guide future researchers.

## Data Availability

N/A.
